# Long-lasting insecticidal nets and indoor residual spraying may not be sufficient to eliminate malaria in a low malaria incidence area: results from a cluster randomized controlled trial in Ethiopia

**DOI:** 10.1186/s12936-019-2775-1

**Published:** 2019-04-18

**Authors:** Eskindir Loha, Wakgari Deressa, Taye Gari, Meshesha Balkew, Oljira Kenea, Tarekegn Solomon, Alemayehu Hailu, Bjarne Robberstad, Meselech Assegid, Hans J. Overgaard, Bernt Lindtjørn

**Affiliations:** 1 School of Public Health, Hawassa University, Hawassa, Ethiopia; 2 Department of Preventive Medicine, School of Public Health, Addis Ababa University, Addis Ababa, Ethiopia; 3 Aklilu Lemma Institute of Pathobiology, Addis Ababa University, Addis Ababa, Ethiopia; 4 Centre for International Health, University of Bergen, Bergen, Norway; 5 Norwegian University of Life Sciences, Ås, Norway

**Keywords:** Cluster-randomized controlled trial, Incidence, Anaemia, Indoor residual spraying, Long-lasting insecticidal nets, Malaria, Vector control, Ethiopia

## Abstract

**Background:**

Conflicting results exist on the added benefit of combining long-lasting insecticidal nets (LLINs) with indoor residual spraying (IRS) to control malaria infection. The main study objective was to evaluate whether the combined use of LLINs and IRS with propoxur provides additional protection against *Plasmodium falciparum* and/or *Plasmodium vivax* among all age groups compared to LLINs or IRS alone.

**Methods:**

This cluster-randomized, controlled trial was conducted in the Rift Valley area of Ethiopia from September 2014 to January 2017 (121 weeks); 44 villages were allocated to each of four study arms: LLIN + IRS, IRS, LLIN, and control. Each week, 6071 households with 34,548 persons were surveyed by active and passive case detection for clinical malaria. Primary endpoints were the incidence of clinical malaria and anaemia prevalence.

**Results:**

During the study, 1183 malaria episodes were identified, of which 55.1% were *P. falciparum* and 25.3% were *P. vivax*, and 19.6% were mixed infections of *P. falciparum* and *P. vivax*. The overall malaria incidence was 16.5 per 1000 person-years of observation time (PYO), and similar in the four arms with 17.2 per 1000 PYO in the LLIN + IRS arm, 16.1 in LLIN, 17.0 in IRS, and 15.6 in the control arm. There was no significant difference in risk of anaemia among the trial arms.

**Conclusions:**

The clinical malaria incidence and anaemia prevalence were similar in the four study groups. In areas with low malaria incidence, using LLINs and IRS in combination or alone may not eliminate malaria. Complementary interventions that reduce residual malaria transmission should be explored in addition to LLINs and IRS to further reduce malaria transmission in such settings.

*Trial registration* PACTR201411000882128 (08 September 2014)

**Electronic supplementary material:**

The online version of this article (10.1186/s12936-019-2775-1) contains supplementary material, which is available to authorized users.

## Background

Despite remarkable achievements in the fight against malaria over the last decade, the World Health Organization (WHO) recommends universal coverage of populations at risk with long-lasting insecticidal nets (LLINs) and targeted indoor residual spraying (IRS) with an insecticide for the control of malaria [1, 2]. Both LLINs and IRS have been shown to be effective in reducing malaria transmission when applied independently [3]. In an effort to accelerate the control and ultimate elimination of malaria, IRS in combination with LLINs has been deployed in some countries [4], and the available evidence from large surveys [5], cohort studies [6], and a randomized trial [7] suggests that the joint intervention of LLINs and IRS should be scaled up and that the combined effect of these interventions should be further evaluated.

Reviews by Pluess et al. in 2009 and WHO in early 2014 documented that historical and programme documentation had clearly established the impact of IRS [3, 8]. However, the number of high-quality trials was too few to quantify the size of effect in different transmission settings. Evidence from randomized comparisons of IRS vs no IRS had confirmed that IRS reduced malaria incidence in unstable malaria settings. Some limited data suggested that LLIN gives better protection than IRS in unstable areas, and these reviews together with modelling efforts, recommended that more trials were needed to compare the effects of LLINs with IRS, as well as to quantify their combined effects.

Despite an increasing interest in the simultaneous use of both interventions, no clear guidelines existed at the start of this study on how these interventions should be combined [9]. At the same time, there is a paucity of evidence concerning whether their combined use is more effective in reducing the incidence of malaria than using either intervention alone [3, 10, 11]. Some non-randomized observational studies and mathematical modelling exercises indicate modest effectiveness or conflicting results when combining interventions for malaria reduction compared to either intervention alone [6, 12–14]. Consequently, it is difficult to draw any conclusions regarding whether the combination of IRS and LLINs is beneficial against malaria compared to one of the interventions alone.

Recent reviews indicated that only a few of the published randomized controlled trials showed additional protection against fighting malaria when the use of LLINs was combined with IRS, compared to either method alone [9, 11]. A multi-intervention trial in Benin reported no reduction in clinical malaria in children under the age of 5 years from houses sprayed with bendiocarb in combination with LLINs, compared to children in houses with LLINs alone [15]. Similarly, in The Gambia, a combination of IRS using DDT and universal coverage of LLINs showed no added protection against malaria among children 6 months to 14 years old compared to universal coverage of LLINs alone [16]. By contrast, a recent cluster-randomized controlled trial in Tanzania, where the usage of LLINs was less than 50%, found some evidence of added protection against malaria infection in children 6 months to 14 years from the combination of LLINs and IRS with bendiocarb compared to LLINs alone [7].

The specific objectives of this intervention study were: (1) to determine whether the combined use of LLINs and IRS with propoxur provides additional protection against malaria (*P*. *falciparum* and/or *P*. *vivax*) among all age groups in the study area compared to LLINs or IRS alone; and, (2) determine whether LLINs + IRS improves haemoglobin (Hb) concentration and reduces anaemia among children under 5 years of age compared with children in LLINs or IRS arms alone.

## Methods

This study was conducted to evaluate the effect of LLINs and IRS to prevent malaria in southern Ethiopia, and followed the recommendations of Lines and Kleinschmidt [17]. This report includes a comprehensive assessment of the trial results. In parallel with this study, monitoring of LLIN ownership and use, entomological studies and monitoring of insecticide resistance are published in separate reports [18–22]. The study protocol has been published previously [23].

### Study setting

This study was carried out in the Adami Tullu part of the Adami Tullu-Jiddo-Kombolcha *woreda* (district) in the East Shewa Zone of the Oromia Regional State in Ethiopia. The *woreda* is a local administrative unit in the country, which consists of several *kebeles* (the lowest government administrative unit; *kebele* is further divided into *gares*, or villages). Administratively, the district comprises 48 *kebeles*, each with a population size of approximately 1000 to 5000 people.

The projected population size of the district for 2014 was approximately 173,000 people [23]. The main ethnic group is the Oromo, and the predominant religion is Islam. The majority of the population lives in rural areas in houses made of mud or cement walls and thatched or iron roofs. Local residents primarily depend on farming, livestock rearing, and to a lesser extent, fishing in Lake Zeway, for their subsistence.

In 2014, there was one public and one non-governmental organization hospital, 9 public health centres, and 43 health posts in the district. Each *kebele* has at least one health post staffed by two health extension workers (HEWs) reporting to the health centre.

The peak malaria transmission season in the study area is from September to December, following the rainy season during June to August. *Plasmodium falciparum* and *P. vivax* are the main causes of malaria infection in the area. *Anopheles arabiensis* is the major malaria vector in the district and *An. pharoensis* is considered to have an auxiliary role [24]. A study performed prior to the start of this trial demonstrated that *An. arabiensis* was susceptible to propoxur (a carbamate), but resistant to the pyrethroid insecticides. However, *An. pharoensis* was susceptible to all pyrethroids and carbamates tested [24]. In Ethiopia, LLINs and IRS are applied either simultaneously or separately depending on the local setting.

### Design

This 2 × 2 factorial, cluster-randomized, controlled trial was carried out for 121 weeks from late September 2014 to January 2017. The village (or cluster) constituted the unit of randomization and an equal number of villages were randomized to one of the following four arms: (1) LLIN + IRS; (2) LLIN alone; (3) IRS alone; or, (4) control. The control arm received the routine standard practice of malaria prevention of the Ethiopian Malaria Control and Elimination Programme [25]. The control households would receive new LLINs and IRS spraying when the district health office found it appropriate, but during the study period, no communities in the *woreda* received such additional interventions. All people living in the area were offered malaria diagnosis and treatment, if needed, when presenting at a health institution. The trial was performed as described in the previous protocol [23]. Although the study was planned for 104 weeks follow-up, this was extended to 121 weeks to add one additional malaria transmission season.

### Participants

This trial was conducted in the rural communities of the district. Prior to implementing the intervention and randomizing villages to arms, a baseline survey, mapping and a pilot study were carried out to estimate an optimum sample size [24]. A population survey in the study households was repeated at the start of each year.

### Village inclusion and exclusion criteria

Villages located within 5 km from Lake Zeway or the Bulbula River were included in the study, as preliminary findings indicated that the incidence of malaria was highest in this part of the area [24].

### Participant inclusion and exclusion criteria

All consenting residents of households in all clusters were recruited for the study. Residents and household heads who did not provide informed consent were excluded.

### Randomization and masking

**Fig. 1:**
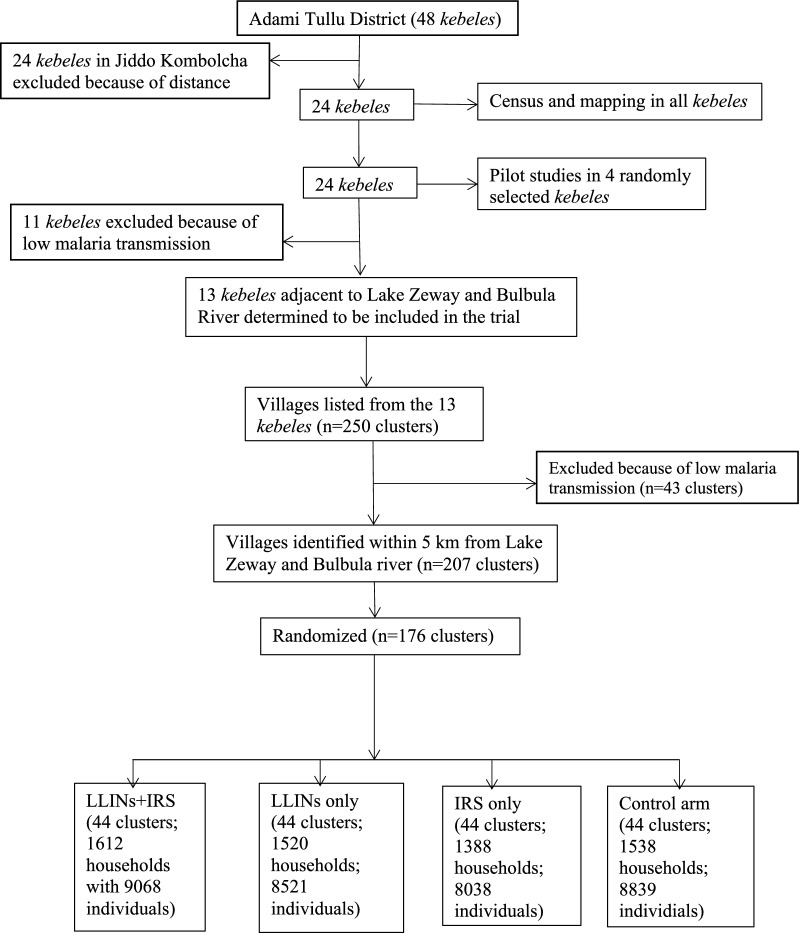
Flow diagram illustrating trial profile

From a total of 48 rural *kebeles* in the Adami Tullu district, 13 *kebeles* adjacent to Lake Zeway and Bulbula River were included in the study. From the total list of the clusters in 13 *kebeles*, 207 were included in the sampling frame, of which 176 were randomly selected (see flowchart, Fig. 1). Randomization was carried out in Bergen, Norway, to prevent selection bias by concealing the allocation sequence from the field researchers assigning villages to intervention groups until the moment of assignment. Thus, a researcher not involved in the study randomly allocated a random number that was used as the seed for the computer-generated list of villages using SPSS software. Because of many clusters in each arm, stratification of clusters or restricted randomization was not done. The baseline data collections were carried out before the start of the study in 2014 showed that the study groups were comparable, except for house design (Table 1).

**Table 1:** Baseline characteristics of study clusters at the beginning of the transmission in 2014

	Intervention arms									
	IRS + LLINs	%	LLINs	%	IRS	%	Control	%	Total	%
Number of clusters	44		44		44		44		176	
Number of households	1618		1388		1527		1538		6071	
Population	9104		8038		8567		8839		34,548	
Population per cluster	207		183		195		201		196	
Age group (in years)										
< 5 years	1673	18.4	1528	19.0	1576	18.4	1711	19.4	6488	18.8
5–14 years	2840	31.2	2706	33.7	2832	33.1	2758	31.2	11,136	32.2
15 years and older	4591	50.4	3804	47.3	4159	48.5	4370	49.4	16,924	49.0
Total	9104		8038		8567		8839		34,548	
Gender										
Male	4612	50.7	4006	49.8	4312	50.3	4397	49.7	17,327	50.2
Female	4492	49.3	4032	50.2	4255	49.7	4442	50.3	17,221	49.8
Total	9104		8038		8567		8839		34,548	
Ethnicity										
Oromo	8819	96.9	7550	93.9	7627	89.0	7821	88.5	31,817	92.1
Amhara	15	0.2	34	0.4	34	0.4	126	1.4	209	0.6
Gurage	8	0.1	21	0.3	164	1.9	65	0.7	258	0.7
Others	262	2.9	433	5.4	742	8.7	827	9.4	2264	6.6
Total	9104		8038		8567		8839		34,548	
Main roof material										
Thatch/leaf	4353	47.8	3863	48.1	4035	47.1	3774	42.7	16,025	46.4
Corrugated iron	4726	51.9	4133	51.4	4484	52.3	5055	57.2	18,398	53.3
Cement/concrete	25	0.3	42	0.5	48	0.6	10	0.1	125	0.4
Total	9104		8038		8567		8839		34,548	
Religion										
Orthodox Christian	717	7.9	918	11.4	921	10.8	937	10.6	3493	10.1
Muslim	8275	90.9	6923	86.1	7437	86.8	7547	85.4	30,182	87.4
Protestant Christian	102	1.1	182	2.3	199	2.3	322	3.6	805	2.3
Other	10	0.1	15	0.2	10	0.1	33	0.4	68	0.2
Total	9104		8038		8567		8839		34,548	
Education status										
Illiterate	5187	57.0	4625	57.5	5043	58.9	4897	55.4	19,752	57.2
Read and write only	866	9.5	918	11.4	1139	13.3	815	9.2	3738	10.8
Primary	2206	24.2	1888	23.5	1820	21.2	2241	25.4	8155	23.6
Secondary and above	845	9.3	607	7.6	565	6.6	886	10.0	2903	8.4
Total	9104		8038		8567		8839		34,548	
Socio-economic status										
Lower class	2887	31.7	3171	39.5	2987	34.9	2414	27.3	11,459	33.2
Middle class	3084	33.9	2587	32.2	2754	32.1	3153	35.7	11,578	33.5
Upper class	3133	34.4	2280	28.4	2826	33.0	3272	37.0	11,511	33.3
Total	9104		8038		8567		8839		34,548	

**Fig. 2:**
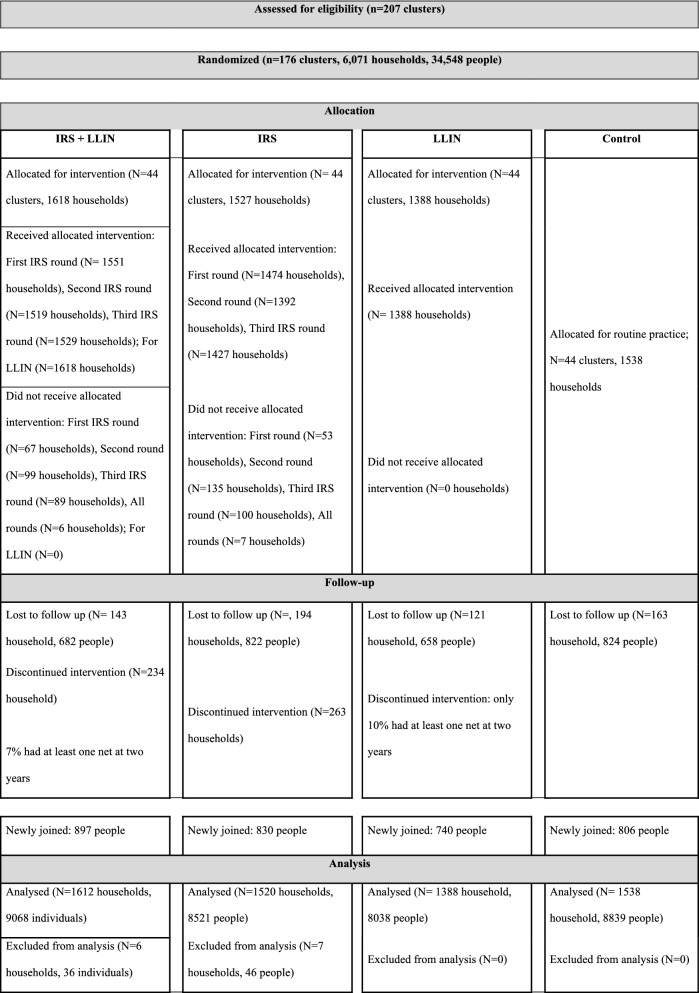
Flow diagram illustrating follow up of trial participants

Due to the nature of the interventions, blinding of the study participants was not possible. Forty-four clusters were assigned to each of the intervention groups. Figure 2 provides information about the interventions in each of the groups, follow-up information, and participants included in the analysis. The LLIN use coverage before the intervention was 11%, and no household had received IRS spraying the year prior to the study.

### Interventions

#### Long-lasting insecticidal nets

The LLINs distributed for this trial were PermaNet 2.0 rectangular, 100 deniers, light blue, large size (160 cm width × 180 cm length × 150 cm height) purchased in June 2014 from the Vestergaard Frandsen Group SA (Vestergaard Frandsen, Lausanne, Switzerland). All households in the IRS + LLIN and LLIN alone arms received new LLINs free of charge at the beginning of the intervention regardless of previous ownership, with householders maintaining their existing nets at the time of distribution. The number of new LLINs distributed to each household was based on the household size recommended by national malaria guidelines, i.e., one net for a family of 1–2, two nets for a family of 3–5, three nets for a family of 6–7, and four nets for a family of ≥ 8 persons [25].

In advance of the LLIN distribution, all village residents were informed about the distribution of the nets through house-to-house visits, village leaders and community elders. Households not receiving nets during the first distribution, received nets later. Education about and a demonstration of how to use LLINs were given to the recipients by trained field staff and selected village residents (Fig. 2).

All study participants were followed on a weekly basis for 121 weeks, from October 2014 to January 2017. All study participants were followed until the end of the study or until they were lost to follow-up. Newcomers (individuals who joined a cohort as new household members) and newborns during the study period were included in the study (Fig. 2). A cross-sectional survey was carried out at the 110th week post-distribution to assess LLIN ownership among all households that received LLINs at baseline and to validate the results of LLIN use.

Weekly home visits were carried out to record the LLIN use of the study participants. Each week, the heads of households or family members aged more than 18 years were asked whether any household members used an LLIN the night before the day of the interview. The names and codes of the individuals who used the LLIN were recorded. If the visited houses were closed, or if heads of households or family members aged more than 18 years were absent, the data collectors visited the house at least three more times within the same week. If one or more or all of the household members had left the study area during the study period, the individuals were considered lost to follow-up. In subsequent follow-ups, the households were visited on the same day of the week to maintain a seven-day gap between visits. The visits were carried out early in the morning to observe whether the LLINs were hung in the sleeping space. For the LLIN ownership survey, respondents were asked if they had useable LLINs in their household. The presence of usable LLINs was verified in the visited household by observation. If the LLINs were lost, the reasons for the loss were asked.

#### Indoor residual spraying (IRS)

Indoor residual spraying with propoxur was carried out three times (September 2014, July 2015, July 2016) during the study period in the LLIN + IRS and IRS alone arms. Spraying was done once a year prior to the peak transmission season, following the national spraying operation guidelines [25].

A 6-day spray operation training was given for locally recruited spray personnel and supervisors. The spraying teams were organized into teams of four spray personnel and a porter, and supervised by a squad leader. Approximately 12 houses were sprayed by each spray operator per day using an 8-l Hudson X-pert (HD Hudson Manufacturing Co, Chicago, IL, USA). Prior to spraying, community sensitization was performed to inform residents about the safety, purpose and time of spraying. IRS operation was performed using propoxur (isopropoxy-phenyl methylcarbamate) purchased from the state-owned Adami Tullu Pesticide Processing Share Company located in the study district.

#### Study endpoints

The primary outcome measure was malaria incidence determined by the detection of *P. falciparum* or *P. vivax* by rapid diagnosis tests (RDTs; CareStart Malaria *Pf/Pv* combo test; Access Bio Inc, NJ, USA) in patients with a fever or having a history of fever within the previous 48 h upon arrival to health posts by active and passive case detection (see “Data collection methods” for details). The other main outcome was anaemia and Hb concentration in children under the age of 5 years which was measured using a portable photometer (Hb310 analyser, HemoCue^®^ AB, Angelhom, Sweden) at the end of each transmission season.

### Sample size

#### Malaria incidence and anaemia prevalence

The sample size was calculated based on earlier model studies, and assuming that the two interventions would provide protection independently of each other by about 40–50%, assuming an additional effect of IRS and LLIN combination of 25% [7, 10, 26]. The sample size calculations were based on epidemiological data collected in a baseline pilot study in villages adjacent to Lake Zeway during September to December, 2013 [24]. The sample size for the primary endpoint, i.e., the incidence of malaria, was calculated using methods for cluster randomized trials that take into account the intracluster correlation coefficient (ICC), incidence rate, expected effect, and power of the study [27]. A baseline malaria incidence rate of 7.85 per 10,000 person-weeks, or 40.8 per 1000 person years (PY) was used, and the coefficient of variation between clusters within each group of k = 0.27 was used for the sample size estimation [24]. In the study, 176 of 207 clusters living within a distance of 5 km from Lake Zeway were randomly selected [23]. These selected villages had 6071 households with approximately 196 people per cluster were followed for 121 weeks, achieving a 90% power to detect a 25% reduction in the malaria incidence rate in the IRS + LLIN arm compared to LLINs alone or the IRS-only arm, using a two-sided 5% significance level.

Some 6071 households with 34,548 people were included the trial (Fig. 1). The proposed sample size had the power to detect a mean difference between the study arms of 0.5 mg/ml Hb concentration in children under the age of 5 years.

### Data collection methods

#### Epidemiological data collection

Active and passive case detection was done to diagnose malaria cases at the health posts throughout the trial using RDTs. Through weekly household visits, study participants with a fever or having a history of fever within the previous 48 h were given numbered identification cards and encouraged to present to the nearest health posts for testing and treatment. All persons with possible malaria were checked if they had actually visited the health post. In addition, the health centres and the hospital were regularly visited to find malaria cases that could have visited these health facilities without reporting to the field workers. Very few such cases were found, but any person from the study villages treated for malaria at a health centre or hospital in the district was included in the study.

Individuals who were found to be positive for *P. falciparum* by RDT were given artemether–lumefantrine [(AL) Coartem^®^, Novartis, Basel, Switzerland] two times a day for 3 days based on body weight, according to national guidelines [25]. AL is a fixed dose combination of 20 mg of artemether plus 120 mg of lumefantrine. Persons with *P. vivax* infections were treated with chloroquine, 25 mg/kg for 3 days (10 mg base per kg on days 1 and 2, and 5 mg base per kg on day 3). Treatment of other conditions was performed in accordance with national guidelines, or referred to higher-level health facilities. Patients with severe illness at the time of visit (from malaria or other causes) were referred to the nearest health facility.

The Hb concentration was measured in children 6–59 months old in the study households at three time points during the study (December 2014–2016, at end of each year’s main malaria transmission season) to assess the prevalence of anaemia. Through house-to-house visits, a single finger-prick sample was taken from each child, and height and weight were measured. Children with Hb values less than 11 g/dl were defined as anaemic.

#### Validation study

To validate the weekly incidence data, a community-based malaria prevalence survey was done on a randomly selected sample of households taking part from each arm of the trial during the main transmission season in November 2015 among all age groups. All household members were eligible and volunteered to be included in the study. The heads of the households were interviewed using a pre-tested structured questionnaire, and tested individuals for malaria parasites using RDT. In this survey, 4450 persons were included, and 0.46% (21 persons) of those who volunteered to contribute had malaria as assessed by RDT. There was no significant difference among the intervention arms, and the prevalence at week 57 was similar to the number of cases reported during the same week by the malaria incidence assessment.

### Data management

The data collection was done using standardized paper-based forms and questionnaires according to standardized operating procedures. Data were entered by trained data entry clerks and verified by range and consistency checks, and data cleaning was performed weekly. Any discrepancies were corrected by cross-checking against the corresponding original forms and subsequently amended in the final dataset.

To minimize any loss to follow-up, all residents were followed and recorded if they moved out of the trial area or moved from one cluster to another cluster with a different intervention. For residents or respondents who were present at the time of the visit by project staff, basic information about dates and reasons for absence were recorded from other community members, such as friends or neighbours.

### Analysis

The primary health outcome measure was malaria incidence determined by the detection of *P. falciparum* or *P. vivax* using RDTs. All analyses were done on an intention-to-treat basis, regardless of whether the individual household members used LLINs, IRS or neither. All analyses were conducted using Stata version 13 (Stata Corp LP, College Station, TX, USA). Outcomes were compared between study arms.

To control for potential confounding factors, the clustering effect of villages and the effect of repeated measurement in the same individual and individual-level covariates (such as age, gender, LLIN use) were taken into consideration during the analysis. Building materials (roof type) is another potential confounding factor, and was also adjusted for in the regression analysis, which was estimated by a proportional hazards model. For ease of analysis, corrugated iron was merged with cement/concrete roof type. Though the main analysis plan was intention-to-treat, considering known protective effect of LLINs, there was a need to see if the rate of malaria infection for an average LLIN user was different from non-user. Thus, to determine if LLINs provided individual-level protection against malaria, a generalized estimating equation with Poisson log linear model was used to adjust for within-cluster correlation of measurements. Principal component analysis was used to construct a wealth index, as has been described before [28].

SatScan v9.1.1 (http://www.satscan.org/) software was used for spatial and space–time statistical analysis, to identify statistically significant retrospective space–time malaria clusters.

### Ethical approval

The study was approved by the Institutional Review Board (IRB) of the College of Health Sciences at Addis Ababa University, the Ministry of Science and Technology, Ethiopia (ref: 3.10/446/06) and the Regional Committee for Medical and Health Research Ethics, Western Norway (ref: 2013/986/REK Vest).

This study contains a control group, which did not receive any additional interventions except for the routine malaria work carried out by the district health office. The three ethical review boards accepted that such a group was included provided that the malaria incidence was followed closely, and if malaria incidence was not higher than expected. The study regularly monitored the malaria incidence in all four groups throughout the study, and the research did not observe higher incidences in the control group, nor any epidemics.

### Community consultation and sensitization

Prior to the implementation of interventions, a consultative workshop and several meetings were held to explain the objectives, *kebele* selection and randomization, implementation procedures, and expected outcomes of the trial to the communities, with representatives from the Oromia Regional Health Bureau, the East Shewa Zone Health Department, the Adami Tullu District Health Office and the District Administration. Study communities were sensitized prior to randomization through meetings and discussions with community leaders, *kebeles*, village leaders, and community elders.

### Information and informed consent

Verbal informed consent to participate in the study was obtained from the study participants and from parents or guardians for children under 18 years old using the local *Afan Oromo* language. Verbal consent was used because many of the participants could not read and write. This consenting procedure was approved by the three ethical committees. Information sheets were provided about the purpose of the study, and the participants were informed that involvement in the study was voluntary and that they had the right to withdraw at any time regardless of reason. At each data collection, verbal consent was obtained from all study participants, and verbal assent was obtained from parents or guardians for children using the local language. Assurance was given that a refusal to participate in this study would not affect their access to services at the health posts in the study villages in the community. After completion of the study, households in the IRS and control groups received LLINs according to the national guidelines for bed net distribution [25].

### Timelines of activities

Ethical approval and pilot study were conducted in 2013, followed by trial registration, actual intervention and outcome measurement (see Additional file 1: Figure S1).

## Results

### Intervention coverage

Baseline data collections done before the start of the study in 2014 showed that the study groups were comparable, except for house design (Table 1). More households in the control group (57.2 vs 42.8%; χ^2^ = 69.4, P < 0.001) had corrugated iron roofs.

The study population consisted of 34,548 people (70,356 PYs of observation) with an average of 196 people per cluster (Table 1). Of these, 6488 (19%) were children under 5 years of age, 11,136 (32%) were between 5 and 14 years, and 16,924 (49%) were older than 15 years of age.

Table 2 provides information about the follow-up of the four intervention arms. With an average of 2.57 nets per household, a total of 3006 households (1618 households in LLIN + IRS arm and 1388 households in LLIN arm) in both arms of the trial received 7740 LLINs (4157 nets in LLINs + IRS and 3583 nets in LLINs only).

**Table 2:** Coverage of the interventions of long-lasting insecticidal nets (LLIN) and indoor residual spraying (IRS) in the study arms at different time periods

	Intervention arms: coverage of interventions				
	IRS + LLINs N = 1618	LLINs N = 1388	IRS N = 1527	Control N = 1538	Total N = 60,781
LLIN ownership (at baseline)	100	100			100
Mean LLIN use during the specified period (%)					
Weeks 1–26	47	49			48
Weeks 26–52	26	27			27
Weeks 53–79	8	6			7
Weeks 79–121	1	1			1
Mean IRS coverage during specified period (%)					
Weeks 1–52	96		97		97
Weeks 53–104	95		92		94
Weeks 105–121	95		94		95

*LLIN* long-lasting insecticidal nets, *IRS* indoor residual spraying; *N* number of households

### Incidence of malaria by study arm

During the 121 weeks from September 2014 to January 2017, there were 1183 malaria episodes, of which 652 (55.1%) were *P. falciparum* infections, 299 (25.3%) were *P. vivax* infections, and 232 (19.6%) were mixed *P. falciparum* and *P. vivax* infections (Table 3); 124 repeated episodes of malaria were diagnosed (Table 3), and the repeated episodes occurred more than 4 weeks after their first episode.

**Table 3:** Malaria incidence rates for the intervention arms for some background variables

	Intervention arm								
	IRS + LLIN			LLIN			IRS		
	Malaria episodes	Person years	Incidence (95% CI)	Malaria episodes	Person years	Incidence (95% CI)	Malaria episodes	Person years	Incidence (95% CI)
Malaria episodes									
First episode (cases)	287	18,376	15.6 (13.9–17.5)	254	16,972	15.0 (13.2–16.8)	261	16,869	15.5 (13.7–17.4)
All malaria episodes	321	18,713	17.2 (15.3–19.1)	278	17,244	16.1 (14.3–18.1)	291	17,153	17.0 (15.1–19.0)
*P. falciparum*	180	18,713	9.6 (8.2–11.0)	173	17,243	10.0 (8.5–11.5)	153	17,154	8.9 (7.5–10.3)
*P. vivax*	86	18,713	4.6 (3.6–5.6)	69	17,243	4.0 (3.1–4.9)	68	17,154	4.0 (3.0–4.9)
Mixed (*Pf *+* Pv*)	57	18,713	3.0 (2.3–3.8)	36	17,243	2.1 (1.4–2.8)	68	17,154	4.0 (3.0–4.9)
Age									
0–5 years	79	3252	24.3 (18.9–29.6)	83	3105	26.7 (21.4–33.0)	54	2951	18.3 (13.9–23.7)
6–15 years	103	5975	17.2 (14.1–20.8)	91	5933	15.3 (12.2–18.5)	83	5840	14.2 (11.2–17.3)
Older than 16 years	139	9481	14.7 (12.4–17.3)	104	8206	12.7 (10.4–15.3)	154	8360	18.4 (15.5–21.3)
Time period									
Week 1–26	83	4071	21.1 (17.0–26.0)	56	3738	15.0 (11.1–19.0)	75	3839	19.5 (15.1–24.0)
Week 26–52	76	4114	18.5 (14.3–22.6)	61	3765	16.2 (12.1–20.3)	54	3789	14.3 (10.5–18.1)
Week 53–79	62	4091	15.2 (11.4–19.0)	59	3786	15.6 (11.6–20.0)	64	3682	17.4 (13.1–21.6)
Weeks 79–121	100	6437	15.5 (12.5–18.6)	102	5954	17.1 (13.8–20.5)	98	5845	16.8 (13.5–20.1)
Clustering									
Cluster area	104	2720	38.2 (31.4–46.1)	126	2735	46.1 (38.5–54.7)	169	3513	48.1 (41.2–55.8)
Non-cluster area	217	15,993	13.6 (11.9–15.5)	152	14,509	10.5 (8.9–12.2)	122	13,639	8.9 (7.5–10.6)
LLIN use									
Over 50%	13	551	23.6 (13.1–39.3)	13	449	29 (16.1–48.3)	0	9	0.0
25–49%	80	5618	14.2 (11.3–17.6)	72	5224	13.8 (10.9–17.3)	7	135	52 (22.7–102.6)
0–24%	228	12,543	18.2 (15.9–20.7)	193	11,571	16.7 (14.5–19.2)	284	17,010	16.7 (14.8–18.7)

	Control			Total		
	Malaria episodes	Person years	Incidence (95% CI)	Malaria episodes	Person years	Incidence (95% CI)
Malaria episodes						
First episode (cases)	257	18,441	13.9 (12.3–15.7)	1059	70,658	15.0 (14.1–15.9)
All malaria episodes	293	18,752	15.6 (13.9–17.5)	1183	71862	16.5 (15.5–17.4)
*P. falciparum*	146	18,752	7.8 (6.5–9.0)	652	71,862	9.1 (8.4–9.8)
*P. vivax*	76	18,752	4.1 (3.1–5.0)	299	71,862	4.2 (3.7–4.6)
Mixed (*Pf *+* Pv*)	71	18,752	3.8 (2.9–4.7)	232	71,862	3.3 (2.8–3.6)
Age						
0–5 years	54	3429	15.7 (11.6–20.0)	270	12,742	21.2 (18.6–23.7)
6–15 years	98	5979	16.4 (13.2–20.0)	375	23,727	15.8 (14.2–17.4)
Older than 16 years	141	9344	15.1 (12.6–17.6)	538	35,393	15.2 (13.9–16.5)
Time period						
Week 1–26	78	4107	19.0 (14.8–23.2)	292	15,755	18.5 (16.4–20.7)
Week 26–52	60	4112	15.0 (11.0–18.3)	251	15,780	16.0 (14.0–18.0)
Week 53–79	71	4 087	17.4 (13.3–21.4)	256	15,646	16.4 (14.4–18.4)
Weeks 79–121	84	6445	13.0 (10.3–16.0)	384	24,681	15.6 (14.0–17.1)
Clustering						
Cluster area	156	3,704	42.1 (35.9–49.1)	484	12,672	38.2 (34.9–41.7)
Non-cluster area	137	15,049	9.1 (7.6–10.7)	575	59,190	9.7 (8.9–10.5)
LLIN use						
Over 50%	0	0	0.0	26	1009	25.8 (17.2–37.2)
25–49%	3	94	32 (8.1–86.9)	162	11,071	14.6 (12.5–17.0)
0–24%	290	18,658	15.5 (13.8–17.4)	995	59,782	16.6 (15.6–17.7)

*LLIN* long-lasting insecticidal nets; *IRS* indoor residual spraying

The overall malaria incidence was 16.5 per 1000 PYs of observation time (PYO). Incidence rates were similar in the four arms with 17.2 per 1000 PYO in the LLIN + IRS arm, 16.1 in the LLIN arm, 17.0 in the IRS arm, and 15.6 in the control arm (Table 3). The incidence of *P falciparum* infection was 9.1 per 1000 PYO (95% CI 8.4–9.8), for *P. vivax* 4.2 (3.7–4.6), and for mixed *P. falciparum* and *P. vivax* infection 3.3 per 1000 PYO (95% CI 2.8–3.6). There was no difference in malaria incidence among the four arms adjusting for roof type. The hazard rate of malaria infection for those living in thatched roofs was 18% higher than in households with corrugated iron roofs (Table 4). The generalized estimating equation (GEE) showed that LLINs did not provide individual protection against malaria infection in the study setting (P = 0.53).

**Table 4:** Proportional hazards model comparing incidence of malaria among the arms, adjusted for roof type

	HR (95% CI)
Arms	
LLIN + IRS	1
LLIN only	0.97 (0.82–1.15)
IRS only	1.01 (0.85–1.19)
Routine	0.92 (0.78–1.08)
Type of house roof	
Thatched/leaf	1.18 (1.04–1.33)**
Corrugated iron and cement/concrete^a^	1

*LLIN* long-lasting insecticidal nets; *IRS* indoor residual spraying

** < 0.01

^a^There were only 125 individuals living under a cement/concrete roof

Of the 1059 households with first episodes of malaria, 484 episodes occurred in areas with malaria clustering. The incidence of malaria in the clustered areas was 38.2 per 1000 PYO, and higher than 9.2 per 1000 PYO in the non-clustered areas; the incidence risk ratio (IRR) was 3.93 (95% CI 3.48–4.38). However, the IRR between the intervention groups in the clustered areas were similar.

### Malaria and anaemia

The prevalence of anaemia was 28.2% (95% CI 26.6–29.8) in 2014 and increased to 36.8% (95% CI 35.1–38.5) in 2015, and fell to 29.8% (95% CI 28.2–31.4) at the end of the study. There was no significant difference in risk of anaemia among the trial arms (Table 5).

**Table 5:** Prevalence of anaemia during the three surveys

Surveys	Number of anaemia cases (haemoglobin < 11 g/dl	Mean haemoglobin (g/dl)	Anaemia prevalence	OR (95% CI)
		(95% CI)	Percent (95% CI)	
Survey 1: December 2014				
LLINs + IRS	199	11.74 (11.63–11.85)	26.8 (23.8–30.1)	1
LLINs only	220	11.52 (11.39–11.64)	28.2 (25.5–31.9)	1.09 (0.87–1.37)
IRS only	199	11.54 (11.42–11.68)	29.1 (25.8–32.6)	1.12 (0.89–1.41)
Control arm	223	11.54 (11.42–11.65)	28.3 (25.2–31.6)	1.08 (0.86–1.35)
All	841	11.59 (11.53–11.65)	28.2 (26.6–29.8)	
Survey 2: December 2015				
LLINs + IRS	310	11.13 (11.01–11.24)	38.1 (34.8–41.5)	1
LLINs only	282	11.22 (11.10–11.34)	35.0 (31.8–38.4)	0.88 (0.72–1.07)
IRS only	272	11.20 (11.07–11.32)	38.5 (35.0–42.2)	1.02 (0.83–1.25)
Control arm	287	11.38 (11.27–11.50)	35.8 (32.5–39.2)	0.91 (0.74–1.11)
All	1151	11.23 (11.17–1129)	36.8 (35.1–38.5)	
Survey 3: December 2016				
LLINs + IRS	240	11.58 (11.48–11.69)	29.5 (26.5–32.8)	1
LLINs only	227	11.55 (11.45–11.66)	31.1 (27.8–34.6)	1.08 (0.87–1.34)
IRS only	192	11.62 (11.52–11.73)	28.8 (25.4–32.2)	0.96 (0.77–1.20)
Control	236	11.57 (11.47–11.67)	29.7 (26.7–33.0)	1.01 (0.81–1.25)
All	895	11.58 (11.53–11.63)	29.8 (28.2–31.4)	

## Discussion

The main finding in this study was that LLINs and IRS, alone or in combination, did not reduce malaria incidence to levels feasible for malaria elimination. The average malaria incidence across study arms was 16.5 episodes per 1000 PYO and there were no significant differences between study arms. The potential reasons for these results are discussed below.

In this study, incidence of malaria was low, and the trial did not demonstrate any reduction in malaria incidence in the intervention groups. The study did not document any additional benefit in using the combination of LLINs plus IRS compared with single interventions with a low malaria incidence. However, the entomological results from the study indicate that combining IRS with LLINs reduced *An. arabiensis* densities compared to LLINs alone and to the control group [22].

Despite the fact that the population is representative of the rural population living in similar ecological settings in Ethiopia, the generalizability of the study findings might be affected by the context of the study period. In the years 2015 and 2016, the study area was affected by an unexpected severe drought and food shortages. This may partially also explain the low LLINs use, as the use of bed nets is associated with lower perceived risk of malaria infection [29].

Although the interventions resulted in lower mosquito densities in houses using IRS compared with LLINs and the control arm [22], the current study did not find a similar effect on malaria incidence. The study shows that IRS or LLINs [even at varying degrees of coverage (Table 3)] may not be able to reduce malaria incidence further in areas with a low malaria incidence. The study suggests that using LLINs and IRS alone in areas with low malaria incidence may not be able to substantially reduce malaria incidence or eliminate malaria, as has also been suggested in a recent review and modelling studies [14, 30, 31].

A study performed in Benin could not document any effect of LLINs or the combination of IRS and IRS on the incidence of malaria [15] and neither did a study from The Gambia [16]. A cohort study with a higher incidence carried out in southwest Ethiopia demonstrated that the use of LLINs, although providing individual protection, did not have an effect on incidence, while IRS spraying showed a reduction in malaria incidence [32]. The study results should not be interpreted to indicate that areas with higher incidences would not benefit from such interventions. LLINs and IRS did not have an observable impact in this study which was conducted in an area of low transmission. However, both interventions reduced malaria incidence in southwest Ethiopia and Sudan in areas of higher transmission [31, 32].

The occurrence of malaria is different from other African countries and is characterized by a mixture of *P. falciparum* and *P. vivax* infections, and where *An. arabiensis* is the principal vector.

The impact of residual malaria transmission was mainly driven by outdoor biting and early indoor biting behaviour of *An. arabiensis* [20]. Complementary interventions that reduce the risk of acquiring malaria infections both outdoors and before sleeping hours, such as toxic sugar bait and interventions that reduce the density of mosquitoes that feed on cattle, e.g., ivermectin, should be explored in addition to LLINs and IRS to further reduce malaria transmission in such settings [33].

A strength of this study is that it was followed by regular monitoring of insecticidal susceptibility of *An. arabiensis* [19, 21]. During the trial period, there was no change either in susceptibility to the carbamates or to the pyrethroids of *An. arabiensis* [18, 21]. *Anopheles arabiensis* was resistant to deltamethrin, while *An. pharoensis* remained susceptible to all insecticides [21].

The bio-efficacy of LLINs was acceptable for at least 24 months [18]. Nevertheless, IRS use remained high during the malaria transmission seasons, and *An. arabiensis* was sensitive to propoxur, assessed by doing monthly cone bioassays for at least the malaria transmission periods [21]. The frequency of pyrethroid resistance to *An. arabiensis* remained high (over 90%) and stable throughout the study [18]. The bio-efficacy of nets to insecticide-susceptible insectary colony of *An. arabiensis* was high [18]. Susceptibility to deltamethrin was restored after exposure of *An. arabiensis* to piperonyl butoxide (PBO), implicating the role of mixed function oxidases in the resistance of this insecticide [21]. Recent trials using LLINs with permethrin (a pyrethroid) and pyriproxyfen had increased efficacy compared with LLINs treated with permethrin alone [34], and introducing this new LLIN could be explored in areas where there exists *An. arabiensis*. In another study, the PBO long-lasting insecticidal net and non-pyrethroid indoor residual spraying interventions showed improved control of malaria transmission compared with standard long-lasting insecticidal nets where pyrethroid resistance is prevalent [35]. As a result, WHO has since recommended to increase coverage of PBO long-lasting insecticidal nets.

The study showed a low LLIN ownership after 2 years, and a low LLIN use, despite 100% net coverage at baseline [19]. The use of LLINs was closely monitored through weekly home visits, and this rigorous monitoring gives a more realistic assessment than some cross-sectional surveys [19, 29]. Another study from the same trial population found behavioural, socio-cultural, economic, and ecological conditions, weak education, communication and social support structures were important in understanding and accounting for why a low level of intended use and a widespread misuse and repurposed use [36]. The study highlights the need to design strategies to increase LLIN ownership and use in low malaria transmission setting.

A strength of this study is that it was based on a random selection of villages: typical rural communities in Ethiopia. Moreover, the study included a large sample with high power and an adequate follow-up period. However, as the incidence rate of malaria was lower than expected at the start of the study, this could affect the statistical power of the study. The research was based on a hypothesised effect size of 30–50% reduction in malaria incidence. Using an effect size of 30% between the LLIN + IRS and the Control arm, a sensitivity analysis showed that the statistical power is 82%. In the study, except for housing type and the interventions, the baseline characteristics of the study arms were balanced (Table 1). In addition, quality-checks on the reported malaria cases took place at health posts, health centres and hospitals in the area. It is unlikely that the study would have missed many malaria cases. The method used to find malaria cases was based on active and passive case finding using trained staff and appropriate RDTs to diagnose malaria. As an additional quality check, a prevalence survey at one point demonstrated that the incidence and the prevalence survey provided similar results, which were also comparable to results from the Malaria Indicator Survey in the same period [37].

Another limitation of the study could be the potential spill-over effect between clusters, and such an effect could have diluted any difference in the outcome measure. However, the IRS + LLINs were as effective as IRS alone in reducing densities and human biting rates of *An. arabiensis*, and the effectiveness of the two interventions combined was better than LLINs alone in reducing densities and human biting rates of the vector [22]. Added impact of the combination intervention against malaria infectivity rates of *An. arabiensis* compared to either intervention alone remains unknown and warrants further research and action.

The study shows that malaria infection is a risk factor for anaemia [28], but the prevalence of anaemia was similar in the trial arms (Table 5). Despite the malaria prevention efforts, an unexpected increase in anaemia prevalence was observed during the 1st year of this study, most probably because of increasing rates of stunting during this period with food shortages [28, 38]. The risk of anaemia was high among children with malaria, children from poor families, stunted children, and children under 36 months old [28]. Conducting malaria prevention trials in drought-prone areas may bring challenges, and a broader assessment of causes of anaemia than used may be appropriate in settings similar to those in this trial.

## Conclusions

The clinical malaria incidence and anaemia prevalence were similar in the four study groups. In areas with low malaria incidence, using LLINs and IRS in combination or alone may not eliminate malaria. Complementary interventions that reduce residual malaria transmission should be explored in addition to LLINs and IRS to further reduce malaria transmission in such settings.

## Additional file

**Additional file 1: Figure S1.** Schedule of enrolment, interventions and assessments.

## Abbreviations

GEE: generalized estimating equation
Hb: haemoglobin concentration
LLINs: long-lasting insecticidal nets
IRB: Institutional Review Board
IRS: indoor residual spraying
PBO: piperonyl butoxide
PYO: person-years of observation time
RDT: rapid diagnosis test
WHO: World Health Organization
